# Genome-Wide Study of Drug Resistant *Mycobacterium tuberculosis* and Its Intra-Host Evolution during Treatment

**DOI:** 10.3390/microorganisms10071440

**Published:** 2022-07-17

**Authors:** Denis Lagutkin, Anna Panova, Anatoly Vinokurov, Alexandra Gracheva, Anastasia Samoilova, Irina Vasilyeva

**Affiliations:** National Medical Research Center of Phthisiopulmonology and Infectious Diseases under the Ministry of Health of the Russian Federation, 127994 Moscow, Russia; panovaae@nmrc.ru (A.P.); vinokurovas@nmrc.ru (A.V.); grachevaan@nmrc.ru (A.G.); samoilovaag@nmrc.ru (A.S.); director@nmrc.ru (I.V.)

**Keywords:** tuberculosis, drug resistance, intra-host evolution, whole-genome sequencing, genome-wide association study

## Abstract

The emergence of drug resistant *Mycobacterium tuberculosis* (MTB) strains has become a global public health problem, while, at the same time, there has been development of new antimicrobial agents. The main goals of this study were to determine new variants associated with drug resistance in MTB and to observe which polymorphisms emerge in MTB genomes after anti-tuberculosis treatment. We performed whole-genome sequencing of 152 MTB isolates including 70 isolates as 32 series of pre- and post-treatment MTB. Based on genotypes and phenotypic drug susceptibility, we conducted phylogenetic convergence-based genome-wide association study (GWAS) with streptomycin-, isoniazid-, rifampicin-, ethambutol-, fluoroquinolones-, and aminoglycosides-resistant MTB against susceptible ones. GWAS revealed statistically significant associations of SNPs within *Rv2820c*, *cyp123* and indels in *Rv1269c*, *Rv1907c*, *Rv1883c*, *Rv2407*, *Rv3785* genes with resistant MTB phenotypes. Comparisons of serial isolates showed that treatment induced different patterns of intra-host evolution. We found indels within *Rv1435c* and *ppsA* that were not lineage-specific. In addition, Beijing-specific polymorphisms within *Rv0036c*, *Rv0678*, *Rv3433c*, and *dop* genes were detected in post-treatment isolates. The appearance of *Rv3785* frameshift insertion in 2 post-treatment strains compared to pre-treatment was also observed. We propose that the insertion within *Rv3785*, which was a GWAS hit, might affect cell wall biosynthesis and probably mediates a compensatory mechanism in response to treatment. These results may shed light on the mechanisms of MTB adaptation to chemotherapy and drug resistance formation.

## 1. Introduction

The spread of *Mycobacterium tuberculosis* (MTB) remains a global public health concern. In recent years, drug-resistant tuberculosis in the world has been one of the main reasons for insufficient improvement of epidemiological indicators. Worldwide, more than 150,000 people with multidrug-resistant tuberculosis (MDR-TB) were enrolled for treatment in 2020, and, despite the steady increase compared to 2012, treatment success rate of MDR-TB was 59% in 2018. Furthermore, more than 25,000 people with pre-extensively and extensively drug-resistant TB (pre-XDR- and XDR-TB) were detected in 2020 [1,2].

For the early detection of drug resistance, it is necessary to have test systems that can quickly determine genotypic factors of reduced susceptibility. However, developing such tests implies the identification of as many loci as possible associated with phenotypic resistance. Genome-wide association study (GWAS) is a powerful approach for searching for associations between phenotypic features and genetic polymorphisms. Initially developed for the human genome [3], GWAS has become a useful tool for the analysis of variants related to virulence and drug resistance in bacteria [4]. However, first attempts to implement bacterial GWAS using known tools indicated that, although the software for human GWAS could identify many variants associated with drug resistance, it also led to false positives owing to confounding from population structure [5]. To evade this limitation, Farhat et al. developed another approach called PhyC (phylogenetic convergence test) [6], which utilizes phylogenetic trees to identify variants under recent convergent evolution. Many resistance-associated variants identified by PhyC were the same as those found by PLINK, but the level of confounding from population structure was reduced. Another noticeable advantage of bacterial GWAS through PhyC is the ability to work with a relatively small sample size. For instance, to date, the most successful study of virulence was of 90 *Staphylococcus aureus* samples [7]. In our work, we used the PhyC-based GWAS approach to search for novel resistance-associated variants using whole-genome sequences of 152 MTB clinical isolates.

Formation of drug resistance is the result of long-term microevolution in the body of a person receiving treatment [8]. Since the development of resistance usually has a fitness cost, compensatory mutations represent another direction of this microevolution [9,10,11]. Furthermore, the bacterial cell tolerates metabolic stress in the presence of drugs, which can cause mutations associated with metabolic adaptation [12,13]. For a more detailed understanding of the mechanisms of drug resistance, it is necessary to investigate the treatment-induced intra-host evolution of MTB. Previous studies in this area have included only one or a few pairs or series of MTB isolated from the same patient at different time points [14,15,16,17,18]. In this study, we examined the effect of anti-tuberculosis drug treatment on the genomes of 32 MTB strains by performing whole genome sequencing of 32 series of isolates obtained before and after treatment. We also tried to clarify whether anti-tuberculosis treatment induced genotype transitions within loci identified by GWAS.

## 2. Materials and Methods

### 2.1. Bioethical Requirements

The materials used in the study did not contain the personal data of patients because the labeling of the clinical isolates did not include name, date of birth, address, or other personal information. Under the requirements of the Russian Federation Bioethical Committee, each patient signed an agreement with the hospital consenting to treatment and laboratory examination.

### 2.2. Sputum Collection and Processing

Sputum samples were collected under standard TB program conditions from patients hospitalized in the National Medical Research Center for Phthisiopulmonology and Infectious Diseases of the Ministry of Health of the Russian Federation and being tested for TB. Specimens were cooled at 2–8 °C until transportation and decontaminated with a BBL MycoPrep System (BD, Franklin Lakes, NJ, USA), according to manufacturer’s protocol. Zero point five milliliters of decontaminated concentrated specimens were inoculated to Löwenstein–Jensen medium (BD, Franklin Lakes, NJ, USA); the same sample volumes were added to Middlebrook 7H9 broth with OADC supplement (BD, Franklin Lakes, NJ, USA). Mycobacterial cells were grown at 37 °C. Non-serial samples were selected based on their phenotypic drug susceptibility. Serial isolates were selected based on the presence of paired isolates from corresponding patients obtained at least 1 month later than the first specimen. The intervals between pre- and post-treatment isolates were 1 to 14 months. Full information about all isolates, including drug susceptibility data and collection dates, is available in Supplementary Table S1.

### 2.3. Antibacterial Susceptibility

Drug susceptibility tests (DSTs) of clinical *M. tuberculosis* strains to isoniazid (INH), rifampicin (RIF), streptomycin (STM), ethambutol (EMB), fluoroquinolones (FQ), amikacin and kanamycin (AMG) were performed by the BACTEC MGIT 960 system (BD, Sparks, MD, USA), according to the manufacturer’s instructions. In this study, DST results were used as phenotype input data for GWAS, and, for convenience, amikacin and kanamycin were combined into the group “aminoglycosides” (AMGs) because of matching susceptibility. Similarly, ofloxacin, moxifloxacin, and levofloxacin were combined into “fluoroquinolones” (FQs). Binary heatmap of DST results was drawn using heatmap R package.

### 2.4. Whole-Genome Sequencing

Isolates were cultured on Löwenstein–Jensen (BD, Franklin Lakes, NJ, USA) slopes, and then mycobacterial genomic DNA was extracted using a DNeasy Blood & Tissue Extraction Kit (Qiagen, Hilden, Germany), following the manufacturer’s protocol. The concentration of DNA was measured with a Qubit fluorometer (ThermoFisher, Waltham, MA, USA). Sequencing library preparation and whole-genome sequencing were performed, as described previously [19]. Raw sequencing reads were deposited to the Sequencing Read Archive under BioProject PRJNA849565.

### 2.5. Genomic Analysis

Quality assessment of all acquired reads was performed with FastQC v.0.11.9 [20]. Variant calling against M. tuberculosis H37Rv (NC_000962) genome was performed using the Snippy pipeline [21]. QualiMap v.2.2.2 was used to check mapping quality [22]. A core SNP alignment was produced with snippy-core v.4.6.0 [21]; SNPs in PE/PPE/PGRS genes, repetitive regions, and mobile elements were partially masked to reduce the false positive rate. Gubbins v.2.4.1 [23] was used to filter out recombinant regions from the alignment. The resulting alignment was cleaned to include only core polymorphic sites with SNP-sites [24]. Cleaned core alignment was used to infer a phylogenetic tree via RAxML v.8.2.12 [25] using the GTRCAT substitution model; the final tree was rooted to the outgroup *Mycobacterium canettii*, which was then removed. The tree was visualized with iTOL online tool [26]. Strains were classified using the pipeline MTBseq v.1.0.4 [27].

### 2.6. GWAS

Filtered (Phred quality ≥ 100, depth ≥ 10, alternate allele count/depth ≥ 0) multi-ssssample vcf-file annotated with snpEff [28] was used as genotypic input, whereas phenotype was presented as a binary matrix. Prewas R package [29] was utilized for data preprocessing—conversion of vcf-file to a binary genotype matrix and ancestral allele reconstruction based on a provided phylogenetic tree. Then hogwash R package [30] was used to perform PhyC-based GWAS of STM-, INH-, RIF-, EMB-, FQ-, and AMG-resistant isolates versus susceptible ones. Based on FDR-corrected *p*-values, the Manhattan plot was built with ggplot2 and ggrepel R packages. We reported all findings that are below a calculated permutation threshold of *p* < 1 × 10^−5^ excluding PE/PPE genes and transposons. All statistical analyses were performed using R software.

### 2.7. Serial Isolates Comparisons

For comparing isolates, one-sample vcf-files were filtered to detect low-frequency variants (Phred quality ≥ 20, depth ≥ 5, alternate allele counts/reference allele counts ≥ 0.1) with bcftools view and intersected serial isolates with each other using bcftools isec [31]. In addition, the same work was performed in parallel with the MTBseq pipeline adjusted to low-frequency variants. Reports of both bcftools isec and MTBseq were carefully checked manually. Processed reports are available in Supplementary Table S2.

## 3. Results

### 3.1. Population Structure, Phylogeny, and Drug Susceptibility Profiling

#### 3.1.1. Strains Classification

The study included 152 *M. tuberculosis* isolates, of which 70 were 32 series of 2–4 samples obtained from the same patients. When classifying isolates, these series were taken as single samples to avoid double counting. Thus, 114 samples were classified, among which 85 (74.56%) belonged to branch 2.2.1 of lineage 2 (Beijing), while the remaining 29 (25.44%) belonged to lineage 4. B0/W148 and Central Asia dominated among the Beijing sublineages (35 and 41 isolates (30.7 and 35.96%), respectively), also Central Asia Outbreak, Ancestral 2, Asian/African, and one unclassified Beijing isolate were revealed. One Beijing isolate belonged to sub-lineage 2.2.2 (Ancestral 1), which is also known as Asian Ancestral [32]. Most of the lineage 4 isolates belonged to the LAM and T-family groups, 11 (9.65%) and 6 (5.26%), respectively, and the remaining samples belonged to the Ural, Haarlem, and Euro-American groups. The population structure is shown in Table 1.

#### 3.1.2. Drug Resistance Profiling

Of 114 samples, 26 (22.8%) samples were pan-susceptible, and 35 (30.7%) were resistant to all five drugs. Of the 26 pan-susceptible strains, 12 belonged to modern Beijing lineages (10 Central Asia, 1 Asian/African and 1 unclassified isolate). The rest of the pan-susceptible samples were a part of lineage 4; 6 belonged to the Euro-American group (4.1 and 4.1.2), 4 to the Euro-American T-family (4.8), 2 samples were classified as Haarlem (4.1.2.1), 1 as Ural (4.2.1), and 1 as LAM (4.3.3). Of 35 samples resistant to all five, 30 belonged to Beijing lineage, the majority (16 of 30) were Beijing B0/W148; therefore, 16 of 35 (45.7%) B0/W148 samples were resistant to all drugs used for DST in this study. Others were classified as Beijing Central Asia, Central Asia Outbreak and Ancestral 2 (12, 1 and 1 samples respectively). The rest of the five polyresistant samples belonged to lineage 4 (4.3.3 LAM). All Beijing B0/W148 strains were resistant at least to STM and INH, and none of them was pan-susceptible.

A total of 83 samples (72.8%) were resistant to two or more drugs. All 72 RIF-resistant strains (63.15%) were also at least resistant to INH and STM. There were 4 samples having isolated resistance to INH, and 8 RIF-susceptible INH-resistant samples had different susceptibility profiles. All AMG-resistant isolates were unsusceptible to at least STM and INH. One sample was susceptible to all drugs except FQ, another isolate carried resistance to FQ and EMB only.

**Figure 1:**
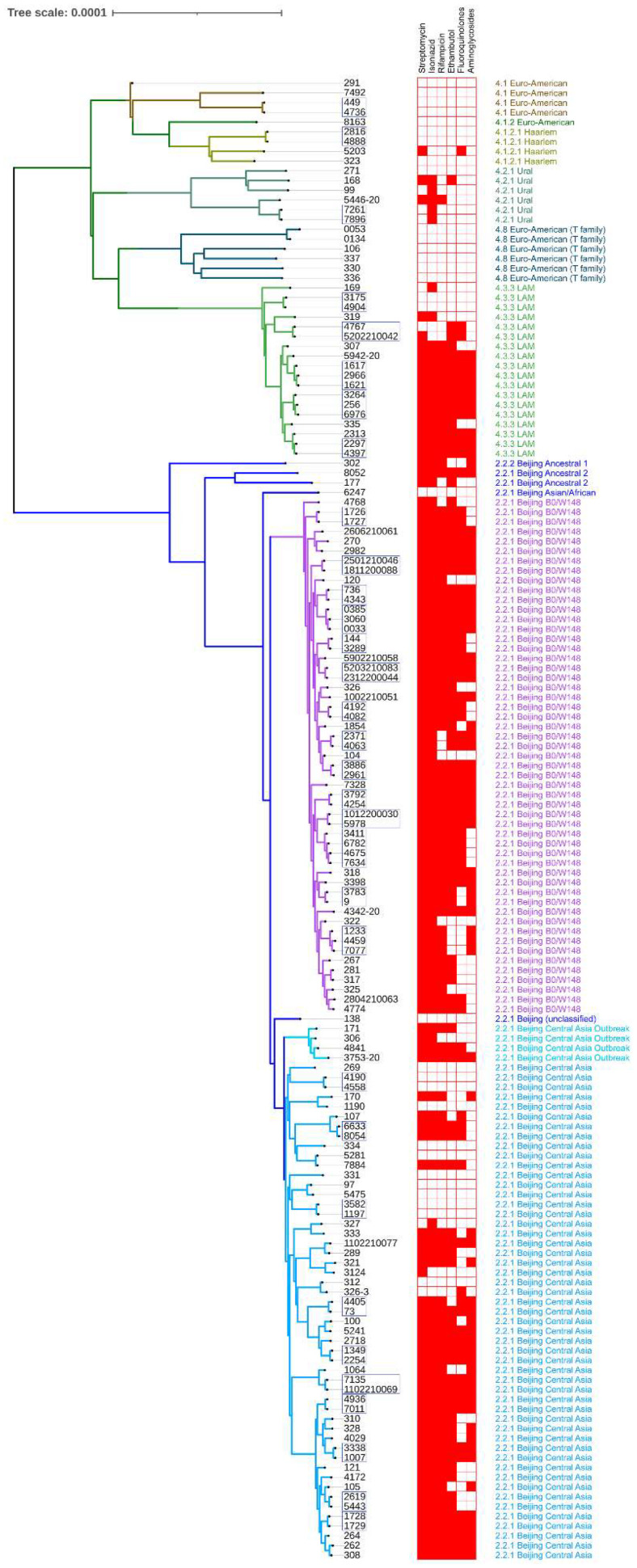
Phylogenetic tree of 152 MTB isolates along with their phenotypic drug resistance profiles and lineages. Branch colors represent lineages or sublineages indicated by these colors on the right. Drug susceptibility profiles are shown as a binary heatmap where red is «resistant» and white is «susceptible». Tree tips corresponding to serial isolates are marked with a blue outline.

Based on 4758 high-quality SNPs, we built a phylogenetic tree, which was rooted on *M. canettii* (not shown) (Figure 1), and added DST data and lineage information.

### 3.2. Convergence-Based Search of Resistance-Associated Polymorphisms

**Figure 2:**
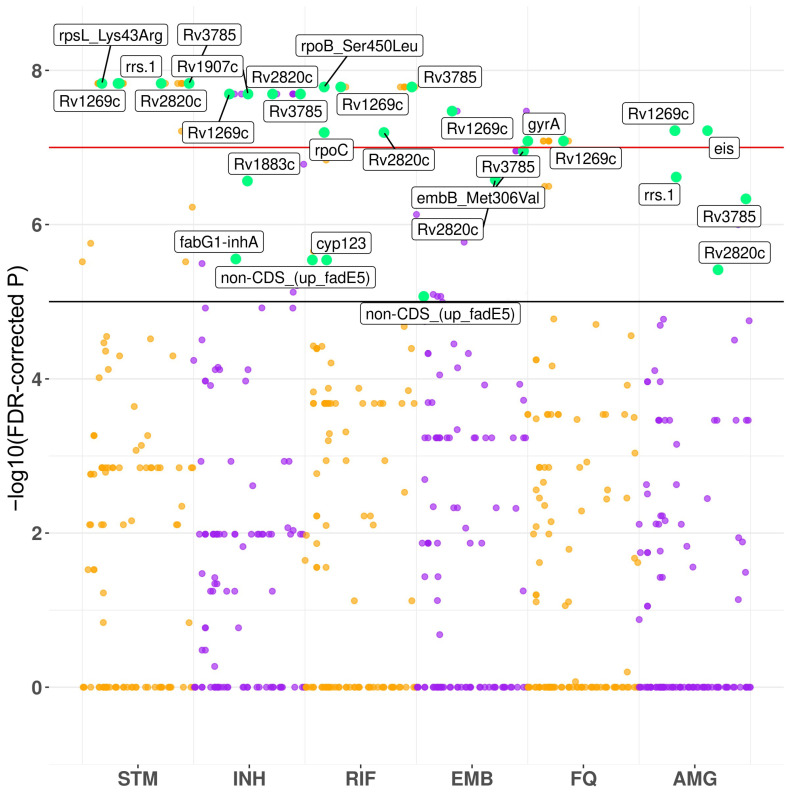
Manhattan plot with PhyC-based GWAS hits for each genotype tested. Green dots show statistically significant hits within and close to coding sequences. Confidence thresholds correspond to *p* = 1 × 10^−5^ and *p* = 1 × 10^−8^.

We performed PhyC-based GWAS of STM-, INH-, RIF-, EMB-, FQ-, and AMG-resistant MTB versus susceptible isolates. Associations with resistant phenotypes were observed within known loci for all drugs: *rpsL*, *rrs* for STM; *fabG1-inhA* promoter region and *katG* for INH; *rpoB* and *rpoC* for RIF; *embB* for EMB; *gyrA* for FQ; *eis* promoter region and *rrs* for AMG. However, GWAS also revealed several other genes, which were not previously described as resistance-associated. Some of these new loci showed association with multiple drug resistance, probably with MDR/XDR phenotype. The most significant associations (Table 2; Figure 2) were observed between all resistant phenotypes and 8-bp insertion in *Rv1269c*. Resistance to all drugs except FQ was associated with nonsynonymous SNP in *Rv2820c* (Lys114Asn). Analysis also identified 1-bp deletion in *Rv2407* locus as significantly related with resistance to STM, INH, and FQ. Besides, 15-bp insertion within *Rv1883c* and 14-bp deletion in *Rv1907c*, which is a part of *katG* operon, were recognized as a signal of association with resistance to INH. Deletion within non-coding region upstream of *fadE5* gene was associated with resistance to RIF and EMB. All isolates that carried this deletion were RIF-resistant in our study. A non-synonymous mutation in *cyp123* (Met192Arg), which encodes cytochrome P450, showed an association signal with STM, INH, and RIF resistance. Finally, 1-bp frameshift insertion within *Rv3785* showed strong association with the resistance to STM, INH, RIF, and AMG.

### 3.3. Serial Isolates Comparisons

A total of 70 samples consisted of 27 series of 2 isolates, 4 series of 3 isolates, and 1 series of 4 isolates from the same patient obtained at different time points during the treatment (Table 3). We compared the polymorphisms of the later and early isolates and found several genes where SNPs or indels appeared in more than one series.

We observed the emergence of 19-22-bp insertions within the *Rv1435c* gene in 12 series and a non-synonymous SNP resulting in an amino acid change Gly119Ala within this locus in 1 series. All of these 13 series were obtained from patients treated with regimens including fluoroquinolones. Polymorphisms in *Rv1435c* were not lineage-specific and occurred in LAM isolates as well as in Beijing samples. In contrast, we observed emerging SNPs leading to Arg255Pro and Ser433Ala in *Rv0036c* and *Rv3433c*, respectively, only among samples belonging to the Beijing lineage. The 4 series related to LAM and Beijing lineages acquired indels of different lengths within *ppsA* gene during the treatment. The *dop* gene became heterogeneous among treated Beijing isolates; in 9 series it acquired SNPs conferring amino acid changes Pro7Arg, Cys18Tyr, and Cys37Tyr. Three post-treatment isolates of FQ-treated Beijing series acquired short insertions within the *Rv0678* gene, which is considered to be related with resistance to bedaquiline; 1 of these 3 also acquired non-synonymous SNP *Rv0678*: Ile16Asn.

We also detected 2 genotype transitions within the *Rv3785* gene (1-bp frameshift insertions), which was the statistically significant GWAS hit associated with STM-, INH-, RIF-, and AMG-resistant phenotypes.

## 4. Discussion

In this study we detected several new SNPs and indels associated with drug resistance in MTB. In addition, we found several divergent loci with emergent polymorphisms, which were probably induced by anti-tuberculosis treatment. Finally, we observed an acquisition of the insertion, which was a GWAS hit, by two treated isolates.

Our study had several limitations. The diversity of treatment regimens and the number of drugs used did not allow us to associate genotype transitions with specific drugs. Many patients started treatment much earlier than we performed our study. The retrospective design of the study caused lack of information about previous treatment regimens and outcomes of current therapy for a significant proportion of patients. The effect of detected variants on the level of drug resistance was not evaluated.

### 4.1. GWAS Hits

The *Rv1269c* gene encodes a secreted protein with an unknown function. It was shown that *Rv1269c* product may damage the host’s mitochondria [33]. Based on a proteomic approach, the study [34] claimed that Rv1269c is more efficiently secreted in modern Beijing isolates because it was significantly more abundant in cell lysates of ancient Beijing isolates, whereas the transcriptional levels were similar for modern and ancient Beijing MTB. We suppose that 8-bp insertion within *Rv1269c*, which was a significant GWAS hit in our study, is rather a phylogenetic marker of modern Beijing sublineages, because it was found in all modern Beijing isolates. It can be speculated that this insertion increases the virulence of these strains through enhancing Rv1269c protein secretion.

The product of the *Rv2407* gene is annotated as ribonuclease Z, an enzyme with tRNA 3’-endonuclease activity, which is essential for 3’-end maturation of some tRNA precursors in prokaryotes [35]. However, we could not find any experimental data confirming Rv2407’s ribonuclease activity. Several studies revealed that Rv2407 is a type III sulfatase with Zn^2+^-dependent metallo-beta-lactamase activity [36,37,38]. The cAMP-receptor protein (CRP)-binding site that was found upstream of *Rv2407* might indicate that expression of this gene is regulated by CRP [39]. CRP-regulated loci are conserved in pathogenic mycobacteria as opposed to non-pathogenic ones, implying the importance of CRP-regulated genes in pathogenesis [39]. An insertion within *Rv2407* was detected in INH-resistant Beijing isolate [40]. Proteomic analysis found *Rv2407* protein in the lungs of guinea pigs infected with MTB 30 days post-infection but not 90 days post-infection [41].

It was previously shown that fadE5 protein takes part in non-specific mechanisms mediating drug resistance [42]. We observed significant association of the deletion upstream of *fadE5* with resistance to RIF and EMB; probably this polymorphism could affect the level of *fadE5* transcription.

*Rv1883c* was more than twofold under-expressed in Δ*sigD* mutants [43]. Vice versa, *Rv1883c* was noticeably overexpressed in MBT cells with inactivated cell division protein FtsZ. Interestingly, the isoniazid resistance-associated gene *inhA* was significantly downregulated in FtsZ-deficient strain [44]. The emergence of non-synonymous SNP Rv1883c: Val73Ala was observed in INH-resistant isolate in the analysis of pre- and post-treatment isolates [11]. The particular function of the *Rv1883c* product is unknown but this locus seems to be associated with INH treatment. This is consistent with our data.

Deletion of 14 nucleotides within *Rv1907c* gene was previously shown as Beijing-specific and probably not conferring INH resistance; however, one INH-resistant strain was described as lacking known mutations conferring resistance to INH and carrying this 14-bp deletion within the *Rv1907c* [45]. This gene is a part of the *furA-katG-Rv1907c* operon, which mediates response to reactive oxygen species [46]. The particular function of *Rv1907c* and its product is unknown.

*Cyp123*: Met192Arg mutation seems to be associated with a polyresistant phenotype in our study but the putative mechanism is unknown. There is some data that *cyp123* is closely interconnected with the cluster of genes responsible for mycolic acid synthesis [47]. Cyp123 is a cytochrome P450, monooxygenase oxidizing a lot of structurally different molecules.

*Rv2820c* was shown to be truncated in Beijing isolates because of large lineage-specific deletion RD207 [48]. The full-size *Rv2820c* gene encodes Csm4 CRISPR protein which is a part of the crRNA-guided effector complex [49]. However, in Beijing strains RD207 covers two CRISPR loci and seven Cas genes (*Rv2814c–Rv2820c*) leading to the truncation of 184 residues from the C-terminus of Csm4 protein. Furthermore, shortened Csm4 of Beijing isolates has a modified C-terminus comprising four changes compared to H37Rv (117-KELAA-119 in H37Rv versus 117-NEPRR-119 in lineage 2 strains) [50]. It is unclear whether modified Csm4 is still able to act as a CRISPR protein; nonetheless, it was demonstrated that truncated Csm4 became a virulence factor which promotes survival in macrophages and affects host immune responses [50,51]. We found truncated modified *Rv2820c* in all Beijing strains including ancestral. Despite the strong signal of association with drug resistance, we suppose that *Rv2820c*: Lys114Asn is the phylogenetic feature of lineage 2. The probable reason for such signal might be a low mapping quality of several bases near the large deletion RD207, which causes incomplete detection of this SNP. Coll et al. observed the association of ethambutol resistance with *Rv2820c*: Lys114Asn [52] but another study suggests that this polymorphism is rather a phylogenetic marker of Beijing lineage. Considering the presence of Lys114Asn within *Rv2820c* of ancestral Beijing strains in our data, the phylogenetic nature of this polymorphism is more likely.

### 4.2. SNPs and Indels Emerged during the Treatment

*Rv0036c* was identified as upregulated at protein level in proteomic analysis of intraphagosomal MDR-TB cells [53]. In addition, its overexpression induced overexpression of *inhA* both in vitro [54] and in vivo [55] and therefore mediated resistance to INH. Functional domain analysis revealed its catalytic activity in cell metabolism or DNA repair steps [53]. This is consistent with another study, where adenylyl- or CoA-transferase activity was shown for Rv0036c [56]. Structural analysis infers that Rv0036c protein is a mycothiol-dependent metalloenzyme with possible dinB-like activity [57]. DinB is a DNA polymerase IV, which confers a mutator phenotype to the cell when the gene product is overexpressed [58]. It is unclear how the change Arg255Pro observed in our comparisons affects the function of Rv0036c protein.

The product of the *dop* gene catalyzes deamidation of prokaryotic ubiquitin-like protein pup, which can then be ligated to a proteasomal substrate and serve degradation signal [59]. In spite of a highly conserved pattern of SNPs in *dop* of pathogenic mycobacteria [60], in serial isolates comparisons we observed non-synonymous variants within codons 7, 18, and 37, different from those which were described as undergoing mutational shifts with high rates [60]. A recent study showed that SNP within 37 codon of *dop* gene was epistatically linked to resistance-associated loci *katG*:315 and *embB*:296 in Beijing isolates [61].

The *ppsA* gene encodes subunit A of phthiocerol polyketide synthase, which takes part in biosynthesis of surface-exposed lipids. Farhat et al. previously identified this gene as resistance-related [6], and its expression was shown to be dramatically upregulated in RIF-resistant strains [62]. However, another study demonstrated that treatment with linezolid (LZD) causes downregulation of *ppsA* expression, which might lead to increase of outer membrane permeability and thus attenuate MTB [63]. All 4 strains carrying new indels within *ppsA* compared to pre-treatment isolates in our study were treated with linezolid.

*Rv1435c* encodes an unknown secreted protein, which was found in culture filtrates [64,65]. This gene includes at least 5 imperfect 21 bp repeats, and we suppose that the 19-22-bp insertion observed within this locus in treated isolates in our study might be a duplication of this repetitive region. *Rv1435c* was shown to be a part of an *Rv2525c*-coregulated gene cluster, which consists of cell wall synthesis proteins and penicillin-binding proteins. It was demonstrated that this cluster is upregulated in INH- and ETH-treated MTB [66]. In addition, SNPs upstream of *Rv1435c* were described as associated with D-cycloserine resistance [67].

*Rv3433c* encodes a putative bifunctional NAD(P)H-hydrate repair enzyme nnr with both hydro-lyase and isomerase activity. This activity allows an ADP-dependent dehydration of S and R forms of NAD(P)HX, heat- or enzyme-damaged NAD(P)H epimers, and, therefore, promotes the recovery of functional NAD(P)H [68]. NAD(P)H-binding site of mycobacterial nnr is located at position 412 (Asp412). Interestingly, position 443, where we observed amino acid change Ser443Ala in post-treatment isolates, is relatively close to NAD(P)H-binding aspartate [69] and, thus, might affect conformational stability of the active site. We checked string-db [70] and found that orthologues of *Rv3433c* are co-expressed with orthologues of *pncA* gene, which encodes enzyme converting prodrug pyrazinamide (PZA) to active pyrazinoic acid and, thus, is a PZA resistance-associated locus. However, all except one post-treatment isolates with emerging *Rv3433c*: Ser443Ala were not treated with PZA. In addition, the absence of the *Rv3433c* gene is associated with tuberculous meningitis through an unknown mechanism [71].

We observed small frameshift insertions within *Rv0678* of 3 treated isolates. In one series, the insertion was accompanied by a non-synonymous SNP, leading to Ile16Asn change. *Rv0678* encodes mmpR5 protein, a marR-like transcriptional repressor, which controls the expression of mmpL (mycobacterial membrane protein large) transporters. These proteins export mycobacterial lipids for cell wall biosynthesis and were also shown to serve as efflux pumps for azoles [72], BDQ and clofazimine [73,74]. In addition to DNA-binding activity, mmpR5 is able to bind fatty acids, but the role of this binding remains poorly understood [75]. Several frameshift insertions within *Rv0678* were described as conferring resistance to BDQ. It was noted that *Rv0678* polymorphisms associated with BQD resistance are widespread, even among MTB isolated from BDQ-naïve patients [76,77]. Such a spread might indicate the non-specific nature of mmpR5-regulated drug efflux. In our study, 2 of 3 post-treatment isolates with *Rv0678* polymorphisms were obtained from patients treated with BDQ; one was BDQ-naïve. Interestingly, 1 of 3 patients (series 4767-5202210042) was initially (before obtaining the first serial isolate) treated with BDQ and LZD, although the isolate was susceptible to first-line drugs. This led to low-frequency frameshift insertion within *Rv0678* of isolate 4767 conferring BDQ resistance, which then disappeared in post-treatment isolate 5202210042. All three patients were treated with FQ.

### 4.3. Insertion of 1 bp within Rv3785 Was Associated with Drug Resistance and Emerged after Anti-TB Treatment in Beijing Isolates

Rv3785 is a putative NADH-dependent epimerase/dehydratase [78], which is located within the mycobacterial arabinogalactan biosynthetic cluster (*Rv3779* to *Rv3809c*). According to a database of known and predicted protein-protein interactions string-db [70], Rv3785 protein is co-expressed with Udp-galactofuranosyl transferase glft1 and ABC-transporter rfbD, which transports lipoarabinomannan and/or arabinogalactan to the cell surface [79]. Thus, the Rv3785 protein may be involved in cell wall biosynthesis. Protein with similar activity participates in exopolysaccharide synthesis in Enterobacteria [80].

We detected the emergence of 1-bp frameshift insertions within the *Rv3785* gene in two serial post-treatment isolates belonging to lineage 2. Meanwhile, this insertion was strongly associated with resistance to STM, ISO, RIF, and AMG in our study. It was found in 18 isolates, all of which belonged to modern Beijing sublineages; 2 of 18 strains, namely, 269 and 334, were pan-susceptible in our DST. Notwithstanding the susceptible phenotype, multiple non-synonymous SNPs were found within *rpoB*, *rpoC*, *katG*, *gyrA*, *embC*, and several other resistance-associated loci of the 269 sample. A STM resistance-associated variant was observed within the *gid* gene of 334 along with SNPs in *gyrA*, *rpoB*, *rpoC*, *embC*, *rpsL*, and other drug-related loci.

The specific mechanisms of Rv3785 protein participation in resistance-associated processes is unknown, and the association of this locus with drug resistance has not previously been described. Considering the putative role of Rv3785 in cell wall biosynthesis, it can be speculated that *Rv3785* polymorphisms are able to inhibit cell wall synthesis to some extent or, vice versa, to intensify this process as an adaptation to stress caused by treatment. In addition, all 18 isolates carrying the insertion within *Rv3785* had mutations in *embB* or *embC* genes, which encode arabinosyltransferases and are located in the same arabinogalactan biosynthetic cluster; thus, the insertion within *Rv3785* might play a compensatory role in *embB-*/*embC*-mutated strains. Anyway, additional research is necessary to determine the specific mechanism of how *Rv3785* mutations can affect drug susceptibility in MTB.

## 5. Conclusions

In this study, we demonstrated several new drug resistance-associated genetic polymorphisms using the PhyC-based GWAS approach. Some of them (within *Rv2820c* and *Rv1269c*) are more likely phylogenetic markers, whereas others (within *Rv2407*, *Rv1907c*, *Rv1883c*, *cyp123*, *Rv3785*, and upstream of *fadE5*) seem to be associated with decreased drug sensitivity or to be drug resistance-driven because of their functions or cellular localization. Furthermore, we studied the microevolution on the intra-host scale during treatment and found some divergent loci, such as *Rv0036c*, *Rv1435c*, *dop*, *ppsA*, *Rv3433c*, and *Rv0678*. These loci acquired SNPs and/or indels in two or more series during therapy. Besides these findings, we showed that 1-bp frameshift insertion within *Rv3785*, which was a statistically significant GWAS hit, is one of such divergent loci and emerged in treated isolates compared to their untreated pairs. However, we were not able to identify specific evolutionary patterns for particular drugs, We did observe some shared features non-specifically emerging upon treatment. The results of our study may provide some insights into new drug resistance mechanisms and compensatory evolution in MTB.

## Figures and Tables

**Table 1:** Lineage and sublineage distribution of MTB strains, *n* = 114.

Lineage	Sublineage	Branch Name	Isolates (%all)	
2	2.2.2	Beijing Ancestral 1	1 (0.88%)	85 (74.56%)
	2.2.1	Beijing Ancestral 2	2 (1.75%)	
	2.2.1	Beijing Asian/Africa 2	1 (0.88%)	
	2.2.1	Beijing Central Asia	41 (35.96%)	
	2.2.1	Beijing Central Asia Outbreak	4 (3.51%)	
	2.2.1	Beijing B0/W148	35 (30.7%)	
	2.2.1	Beijing (unclassified)	1 (0.88%)	
4	4.1	Euro-American	3 (2.63%)	29 (25.44%)
	4.1.2	Euro-American	1 (0.88%)	
	4.1.2.1	Haarlem	3 (2.63%)	
	4.2.1	Ural	5 (4.39%)	
	4.3.3	LAM	11 (9.65%)	
	4.8	Euro-American (T-family)	6 (5.26%)	

**Table 2:** Statistically significant PhyC-based GWAS hits.

**Streptomycin**			**Isoniazid**			**Rifampicin**		
**Position**	**FDR-Corrected *p***	**Gene**	**Position**	**FDR-Corrected *p***	**Gene**	**Position**	**FDR-Corrected *p***	**Gene**
781687	1.48 × 10^−8^	*rpsL*	1418863	2.02 × 10^−8^	*Rv1269c*	761155	1.64 × 10^−8^	*rpoB*
1473246	1.48 × 10^−8^	*rrs*	2153725	2.02 × 10^−8^	*Rv1907c*	1418863	1.64 × 10^−8^	*Rv1269c*
1418863	1.48 × 10^−8^	*Rv1269c*	3127931	2.02 × 10^−8^	*Rv2820c*	4231948	1.64 × 10^−8^	*Rv3785*
3127931	1.48 × 10^−8^	*Rv2820c*	4231948	2.02 × 10^−8^	*Rv3785*	764841	6.39 × 10^−8^	*rpoC*
4231948	1.48 × 10^−8^	*Rv3785*	2133468	2.72 × 10^−7^	*Rv1883c*	3127931	6.39 × 10^−8^	*Rv2820c*
2704884	3.04 × 10^−5^	*Rv2407*	1673425	2.79 × 10^−6^	*fabG1-inhA* promoter	295719	2.88 × 10^−6^	non-CDS
859498	3.42 × 10^−5^	*cyp123*	2704884	1.20 × 10^−5^	*Rv2407*	859498	2.88 × 10^−6^	*cyp123*
			859498	7.58 × 10^−5^	*cyp123*			
			2155168	7.58 × 10^−5^	*katG*			
**Ethambutol**			**Fluoroquinolones**			**Aminoglycosides**		
**Position**	**FDR-Corrected *p***	**Gene**	**Position**	**FDR-Corrected *p***	**Gene**	**Position**	**FDR-Corrected *p***	**Gene**
1418863	3.37 × 10^−8^	*Rv1269c*	7582	8.25 × 10^−8^	*gyrA*	2715342	6.05 × 10^−8^	*eis* promoter
4247429	1.11 × 10^−7^	*embB*	1418863	8.25 × 10^−8^	*Rv1269c*	1418863	6.05 × 10^−8^	*Rv1269c*
3127931	2.62 × 10^−7^	*Rv2820c*	7570	5.33 × 10^−7^	*gyrA*	1473246	2.42 × 10^−7^	*rrs*
295719	8.55 × 10^−6^	non-CDS	2704884	1.97 × 10^−5^	*Rv2407*	4231948	4.64 × 10^−7^	*Rv3785*
						3127931	3.86 × 10^−6^	*Rv2820c*

**Table 3:** Treatment regimens and emerged genetic polymorphisms in isolates from treated patients compared to pre-treatment isolates.

ID	Treatment	Genotype Transitions						
		*Rv1435c*	*Rv0036c*	*Rv0678*	*Rv3433c*	*dop*	*ppsA*	*Rv3785* *
144-3289	Fq Z Cs Lzd Bdq					Cys37Tyr	Ins	
1007-3338	Fq Cap Cs Lzd Bdq		Arg255Pro				Ins + Del	
1197-3582	H E Z Pto							
1726-1727	N/A							
1728-1729	N/A							
2254-1349	R E Z Fq Cap PAS Cs Lzd Trd Amc		Arg255Pro					
2297-4397	N/A							
2371-4063	R Fq Amg Cs Pto				Ser443Ala			
2619-5443	N/A	Ins	Arg255Pro			Pro7Arg		
2816-4888	H R Fq Amg Lzd							
2961-3886	Fq Lzd Pto Bdq							
3175-4904	H R Z Fq	Gly119Ala						
3783-9	Fq Amg PAS Cs Lzd Mrp				Ser443Ala			
3792-4254	Z Fq Lzd Pto Bdq	Ins						
4190-4558	H R E Z				Ser443Ala			
4192-4082	Fq Cap Cs Lzd Pto Bdq	Ins				Cys18Tyr		
4343-736	Fq Amg Lzd Bdq Amc	Ins		Ins	Ser443Ala	Cys18Tyr		
4405-73	E Z Fq Cs Lzd Bdq Mrp	Ins				Pro7Arg		
4736-449	H R E Z							
4767-5202210042	Z Fq Cap Cs PAS Pto	Ins		Ile16Asn + Ins			Ins + Del	
	Fq Cap Cs Pto Lzd Bdq							
	Fq Cap Cs Pto Mrp Amc							
	H R E Cap Pto							
4936-7011	Z Fq Cap Cs Lzd Bdq Mrp							
5978-1012200030	Z Fq Cs Lzd Bdq Im Amc							
6633-8054	Fq Amg Cap Cs Lzd Bdq Mrp Trd	Ins						
7135-1102210069	Z Fq Cap Cs Lzd Bdq Mrp Amc	Ins						Ins
7261-7896	R Z Fq Amg Cs							
1811200088-2501210046	Fq Cs Lzd Bdq Mrp					Cys18Tyr	Ins + Del	
2312200044-5203210083	Fq Lzd Pto Bdq Mrp Amc					Cys37Tyr + Pro7Arg		
0033-3060	N/A	Ins				Cys37Tyr		
3060-0385		Ins				Cys37Tyr		
2966-1617	R E Z Fq Pto							
1617-1621								
4459-7077	Fq Cap PAS Cs Pto	Ins						
7077-1233		Ins			Ser443Ala	Pro7Arg		Ins
3264-6976	Z Fq Cap PAS Cs							
6976-256				Ins				
3411-4675	Z Fq PAS Cs Pto		Arg255Pro					
4675-6782	Fq Amg Cs Lzd Bdq Mrp Amc		Arg255Pro					
6782-7634	Fq Amg Cs Lzd Bdq Mrp Amc	Ins	Arg255Pro	Ins				

* *Rv3785* is the statistically significant GWAS hit. H—isoniazid, R—rifampicin, E—ethambutol, Fq—fluoroquinolones, Amg—aminoglycosides, Cap—capreomycin, Cs—D-cycloserine, PAS—para-aminosalicylic acid, Pto—protionamide, Z—pyrazinamide, Lzd—linezolid, Bdq—bedaquiline, Mrp/Im—meropenem/imipenem, Amc—amoxicilline clavulanate, Trd—terizidone.

## Data Availability

Raw reads from whole-genome sequencing were submitted to the NCBI Sequencing Read Archive database under BioProject PRJNA849565.

## Supplementary Materials

The following supporting information can be downloaded at: https://www.mdpi.com/article/10.3390/microorganisms10071440/s1, Table S1: Phenotypic drug susceptibility testing results, collection dates (where known) and intervals between isolate collection dates for series; Table S2: Comparisons of sequencing data of serial isolates.
